# Reviewed article: Bicalho JMF, Guimarães AA, Oliveira CDL, Lopes ACS. Perceptions of health professionals about the implementation of the Adequate and Healthy Eating Promotion Program in a municipality of the Minas Gerais: a qualitative study. Epidemiol Serv Saude. 2025:34;e20240408

**DOI:** 10.1590/S2237-96222025v34e20240408.b

**Published:** 2025-07-11

**Authors:** Marcia Cristina Dalla Costa

**Affiliations:** UNIOESTE, CCMF

**Table:** 

Rating	Excellent	Good	Average	Below average	Poor
Interest	✓				
Quality			✓		
Originality		✓			
Overall			✓		

**Table:** 

Questionnaire	Yes	No	Not applicable
Does the manuscript contain new and significant information to justify publication?	✓		
Does the Abstract (Summary) clearly and accurately describe the content of the article?	✓		
Is the problem significant and concisely stated?	✓		
Are the methods described comprehensively?		✓	
Are the interpretations and conclusions justified by the results?	✓		
Is adequate reference made to other work in the field?	✓		

## Manuscript Structure

Length of article is: Too long

Number of tables is: Too few

Number of figures is: Too few

Please state any conflict(s) of interest that you have in relation to the review of this paper (state “none” if this is not applicable): None

## Comments

Os comentários e sugestões foram incluídos no arquivo em anexo. 

